# Performance of Pak Universities in Global Science

**DOI:** 10.12669/pjms.37.4.4603

**Published:** 2021

**Authors:** Sultan Ayoub Meo, Shaukat Ali Jawaid

**Affiliations:** 1 Prof. Sultan Ayoub Meo MBBS, Ph.D., M Med Ed (Dundee), FRCP (London), FRCP (Dublin), FRCP (Glasgow) FRCP (Edinburgh) Professor and Consultant, College of Medicine, King Khalid University Hospital, King Saud University, Riyadh, Saudi Arabia. Email: smeo@ksu.edu.sa sultanmeo@hotmail.com; 2 Shaukat Ali Jawaid Chief Editor, Pakistan Journal of Medical Sciences, Karachi, Pakistan Secretary, Eastern Mediterranean Association of Medical Editors (EMAME) E-mail:pjms@pjms.org.pk

The present modern and highly advanced 21’st century is the era of Science and Technology. Universities are the basic birthplace of higher education, research, and innovation, and playing a major role in the performance, prosperity, and economic progress along with sustainable development of the countries.

Presently, the pattern of living standards, environment, health and disease, war and economies, have been completely changed. This change is rotating around the impact of higher education, universities, research institutes, and their technology-based innovative outcomes. Moreover, the current indicators of strong and sustainable economies are dependent on advanced research. The innovative research in science plays a vital role in planning, living standards, excellence of life, economies, and survival of the countries.^1^ The founder of Pakistan, Quaid Azam Muhammad Ali Jinnah once stated that *“Education is the matter of life and death to our nations. The world is moving so fast that if you do not educate yourselves you will be not only completely left behind but will be finished up”.*

At present Pakistan is home to 224 million people^2^, and 224 chartered universities^3^ including 30 medical universities, 176 public and private sector medical and dental schools^4^, 125 engineering, 98 management sciences, and 30 agricultural institutes. Pakistan’s spending on higher education has multiplied since the last two decades; however, the higher education system has continued to deteriorate. The standard of universities in Pakistan has gone down and they are not focusing on what is considered crucial in the standing of a university: research and invention-based learning.

As per the Academic Ranking of World Universities (ARWU) 2020 report^5^, only one private-sector medical university from Pakistan (Aga Khan University, Karachi) ranked in the top 110 universities, unfortunately, none of the remaining universities could achieve a place among the top 500 global universities. However, only four universities, COMSATS University Islamabad achieved a position (517), Quaid Azam University, Islamabad (854), University of Punjab, Lahore (889), and University of Engineering and Technology, Lahore (978). In neighboring country China, 145 universities achieved a place in the ARWU ranking, among them six universities are ranked in the top 100 universities, 71 among top 500 universities; India has 15, and Iran has 11 universities in the ranking list^5^.

Another important area that is closely linked to universities and research institutes and their global standing is the role of academic journals and the visibility of research published in these journals. In Pakistan, there is a total of 179 HEC indexed journals in various academic disciplines. These journals are 58 in Health Sciences; 35 Management Sciences and Economics; 26 Engineering and Technology; Natural Sciences 20, Agriculture 17, Multidisciplinary 15; Social Sciences 04; and Arts and Humanities are 04.^6^

In June 2020, a worldwide notable US-based indexing institute, “Web of Science, Clarivate Analytics” released a journal’s Impact Factor (IF) list for 2019. The impact factor is highly acknowledged in the academic world as a yardstick of a journal’s prestige. From Pakistan, out of 179 only 11 (6.14%)^6^ academic journals achieved a place in Web of Science. Among these journals only one journal crossed the IF of more than 1.0; the remaining journals have an impact factor of around 0.3 - 1.0.^5^

While evaluating the quartile ranking of these journals in a subject category in percentile rank, the quartile ranking of Pakistani journals is: 01 journal in Q2; 02 in Q3; and the remaining 08 journals are in Q4.^5^ Although, some journals are celebrating their silver and golden anniversaries, they could not achieve a rank either in PubMed or in Web of Science. These are major factors that the global science community is not citing the research published in these non-indexed journals.

Worldwide, 12800 science and social sciences journals are indexed in the Web of Science with their IF is ranging from 0.001 to 292.27. The “Cancer Journal for Clinicians USA achieved a top position with Impact Factor 292.27. The other top-ranking journals are the New England Journal of Medicine USA 74.69; The Lancet USA 60.39; Nature UK 42.77; and Science USA 41.84”.^5^ These journals are leading the world in the global IF race.

There are many “science metrics, including Impact Factor, C-index, h-index, Matthew value, Eigen factor, Article influence”, but each of them has its strength and limitation. IF has an impact on the scientific community while taking decisions about where to publish, to promote, whom to hire, and to receive the research grants. There is criticism about the dominancy of IF but still, a large part of the scientific community believes that IF is a powerful tool for the global evaluation of scientific quality. Yet another indicator, Hirsch Index (h-Index) is gaining popularity in the science community; although the h-index is passing in its adolescent age, over time it will be a more powerful metric to measure the scientific credentials.

**Figure:**
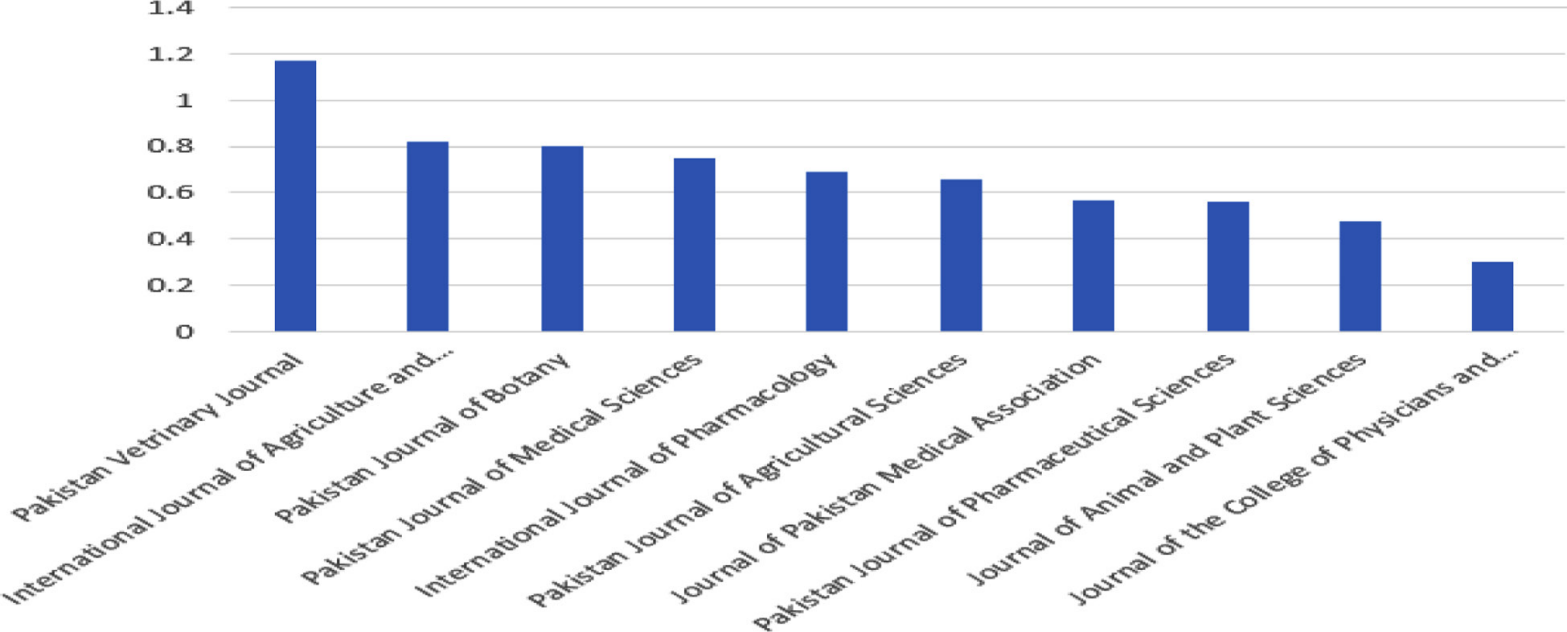
Impact Factor of Pak academic journals.^5^

Since 1947-2020, Pakistan has improved in publishing research articles and has published 193928 articles. In the year 2000, the total number of articles published in Web of Sciences was 641, in 2010 (5595), 2015 (12232), and in the year 2020 (28802)^5^. The quantity is markedly improved but global citations are poor. It shows that the science community is not citing the research published from our country, as researchers and institutes are lacking in producing novel and innovative research. Most of the studies are a repetition of work and questionnaire-based KAP survey studies. The current research is not linked with industries; hence it is not possible to transform the research towards a knowledge-based economy. The universities and research institutes have failed to guide, support, and train the students and faculty towards innovative research.

According to the World Economic Forum Global Competitiveness Report 2019-2,^8^, Pakistan’s rank is 110th out of the total 141 countries. This position is lower than Nepal, (108), Cambodia (106), Bangladesh (105), and close to Ghana 111. In the UN Human Development Index 2021,^9^ Pakistan went down to a rank of 154. These figures are close to countries including Syria (151), Cameroon (153), Uganda (159), etc. This is the failure of the authorities who are responsible for the higher education, research, and innovation road map, along with the university executives who are running the higher education system.

In Pakistan, the criteria for appointments for professors and Vice-chancellors is very poor. The university faculty members easily get promoted from Assistant Professor to Associate and Full Professor based on a postgraduate degree and only 8-10 research articles, though the number has recently been increased by HEC. However, it is the quality of research that should matter instead of the number of papers. In Western Universities, with this required selection criterion, even a junior faculty position of a “lecturer” cannot be achieved. However, the criteria for the appointment of Vice-chancellors of universities is almost similar to a professor. Most of the university Vice-chancellors have postgraduate degrees with 8-10 papers published in a web of science indexed or non-indexed peer-reviewed journals. Higher academic titles, an enriched list of research papers in leading scientific journals, global research visibility, a high h-Index, and an invention background should be required for being appointed as a Vice-Chancellor.

It is time for a change, and to understand the worth of higher education, scientific innovative research, and its impact on socio-economic development and political stability. Pakistan should implement strict policies for the appointment of professors, heads of the research institutes, and Vice-chancellors. It should be based purely on high academic and research credentials, merit, and merit alone. If university professors and VCs have poor academic and research credentials, how can their graduates compete with the world and lead the state towards a knowledge-based economy?

Those selected for the coveted post of Vice-chancellor should be distinguished academicians with a strong record of academic and research excellence with professional competency. The minimum requirements for the selection of vice-chancellors, the applicant should be credited with at least 50 original articles published in the web of science indexed journals, and for half of them, the applicant must be the first and corresponding author. The applicants must have an Impact Factor of a minimum of 50, one thousand citations, 20 h-Index. Those with at least two national or international patents, and two books authored and published by reputable publishers should get preference. Finally, they must have at least five years of administrative experience.

The appointment of administrative positions in academic and research institutes including the chairman, head of the institutes, director, principal, dean, and vice-chancellor should be for 3-4 years. And during the period the progress report must be evaluated and published by the concerned authorities and ministries. The tenure track administrative positions will provide opportunities for other faculty members to join and prove their capabilities. This pathway of administrative positions exists in most American, Canadian, and European universities.

A critical analysis of the current situation in Pakistan will reveal that the political appointment of Governors as Chancellors of the universities appears to be a major hurdle in the progress and development of these academic and research institutions. It is time that we change this and make sure that only academicians in their respective fields with proven academic accomplishments, known for their intellectual integrity are considered for Governor, chancellor. This practice has been followed in many countries in which Chancellors are highly qualified and research-oriented academicians.

We have multiple examples in the various regions. In Asia, Europe, US, universities have known academicians as Chancellors. Then to run and manage the universities, they have vice chancellor, and about half a dozen pro vice chancellors responsible for different areas like faculty affairs, student affairs, education, research and innovation, international collaboration, and administrative services etc. They all form the governing body of the university. Similarly, the Medical Journals Evaluation Committee has some distinguished medical editors as members who guided, helped and assisted the medical journals to improve their standard and quality. But in Pakistan, HEC which unfortunately has no one in the executive position with a medical background is looking after medical education and evaluation of Health Science Journals as well. The solution lies in either creating a separate Division of Medical Education and Health Science Journals within the Higher Education Commission or ideally separating it from HEC and renaming the Ministry of Health as Ministry of Medical Education and Health. It should be headed by renowned academicians.

In Pakistan, brilliant students are always selecting the field of medical, engineering, economic, and management sciences, but these fields are acutely lacking in research and innovation-oriented education. The innovative research provides benefits to society and the economy. Sadly, in Pakistan, faculty members are unaware of innovative research, patents, and their linkage with industries. In this situation, students who graduated from these universities cannot compete with their peers at international levels.

For establishing a research-oriented culture, research must be a part of the curriculum and students should be encouraged to learn, and publish their findings in indexed journals. Moreover, the distinguished editors should be involved to train the faculty as well as students. In Pakistan, unfortunately, in HEC, people with executive positions establishing policies for medical education and evaluation of Health Science Journals do not have a medical background. HEC must facilitate the indexing of academic journals in PubMed and Web of Science to fascinate the science community, enhance research visibility and research culture in the region. HEC should involve the renowned medical editors of Impact Factor Journals in Pakistan to improve the standard of other journals.

We need to work on many fronts, building the professional capacity of the author as well as improving the training of the Editors. Ever since its inception, with meager resources, the Pakistan Association of Medical Editors (PAME) have been organizing workshops on Scientific Writing, Peer Review, Publication Ethics, and training courses for Editors on how to run a successful journal, in collaboration with the Eastern Mediterranean Association of Medical Editors (EMAME). EMAME has also been organizing such workshops in other countries of EMR. More recently, PAME has started a Certificate Course in Medical Editing for Editors at the University of Health Sciences (UHS) Lahore. It is a six months course with two contact sessions of four days each. Two batches have qualified so far of which twenty-three in the first batch and thirty-three in the second batch. 10 The third batch is going to be inducted shortly. It will be followed by Advance Course in Medical Editing, to be followed by two more six months modules after which the successful candidates will get a Master’s degree in Health Journalism. Pakistan has started such a course for the training of faculty and Editors.^11^ ,^12^ It is hoped that it will help in improving the standard of national medical journals and research culture in the country.

Presently, the science-diplomacy nexus is growing all around the world. Pakistan should establish science diplomacy for the better understanding and reinforcing the connections between global academia, research, science, technology, and international affairs to tackle national and global challenges. The academicians and scientists should be given some responsibilities as advisers or diplomats to build international partnerships and influence or represent their nation’s interest^13^.

Once highly qualified, research-oriented, merit-based academicians and scientists lead the universities, and states, they will bring a change and prepare the graduates and state to compete in this highly technology-based advanced world, both at regional and global levels. We must follow the advice of Founder of the Nation Quaid-e-Azam Mohammad Ali Jinnah about education mentioned earlier in this write-up.

It is time for our rulers, health planners, the Higher Education Commission, Chancellors and Vice-chancellors and other higher education degree-awarding institutions to wake up, and implement policies; otherwise, the country will be left far behind in the race of Science and Technology. Education and Research are the key to economic progress and sustainable development. Achieving all the above to some may look like an uphill task but it is achievable if we ensure all academic appointments are based on merit and merit alone.
